# Microregional antitumor activity of a small-molecule hypoxia-inducible factor 1 inhibitor

**DOI:** 10.3892/ijmm.2011.875

**Published:** 2011-12-29

**Authors:** KIYOSHI OKAMOTO, DAISUKE ITO, KAZUKI MIYAZAKI, SAORI WATANABE, OSAMU TOHYAMA, AKIRA YOKOI, YOICHI OZAWA, MAKOTO ASANO, TAKANORI KAWAMURA, YOSHINOBU YAMANE, SATOSHI NAGAO, SETSUO FUNASAKA, JUNICHI KAMATA, YOSHIHIKO KOTAKE, MIKA AOKI, NAOKO TSUKAHARA, YOSHIHARU MIZUI, ISAO TANAKA, KOHEI SAWADA

**Affiliations:** 1 Eisai Co., Ltd., Tsukuba, Ibaraki 300-2635; 2 Genomics-Based Drug Discovery, Graduate School of Comprehensive Human Sciences, University of Tsukuba, Tsukuba, Ibaraki 305-8575, Japan

**Keywords:** hypoxia-inducible factor 1, hypoxia, radiation, small-molecule inhibitor, xenograft model

## Abstract

Hypoxia-inducible factor 1 (HIF-1) activates the transcription of genes that play crucial roles in the adaptation of cancer cells to hypoxia. HIF-1α overexpression has been associated with poor prognosis in patients with various types of cancer. Here, we describe ER-400583-00 as a novel HIF-1 inhibitor. ER-400583-00 suppressed the production of HIF-1α protein in response to hypoxia, with a half-maximal inhibitory concentration value of 3.7 nM in human U251 glioma cells. The oral administration of 100 mg/kg ER-400583-00 to mice bearing U251 tumor xenografts resulted in a rapid suppression of HIF-1α that persisted for 24 h. Immunohistochemical analysis revealed that ER-400583-00 suppressed the proliferation of cancer cells most prominently in areas distal to the region of blood perfusion, where HIF-1α-expressing hypoxic cancer cells were located. These hypoxic cancer cells were resistant to radiation therapy. ER-400583-00 showed a synergistic interaction with radiation therapy in terms of antitumor activity. These data suggest that HIF-1 blockade by small compounds may have therapeutic value in cancer, especially in combination with radiation therapy.

## Introduction

Development of hypoxia in cancer poses a problem, because it renders solid tumors resistant to chemotherapy and radiation therapy (1–3). Tumor hypoxia is mainly caused by insufficient vascularization and increased oxygen consumption as a result of rapid cancer-cell proliferation. It has been reported to have an adverse prognostic impact on various types of cancer, including those of the cervix (4), the head and neck (5), and soft tissue sarcomas (6).

Hypoxia-inducible factor 1 (HIF-1) is a master regulator of hypoxic adaptation (7,8). This transcription factor is a heterodimer of HIF-1α and HIF-1β. The HIF-1β protein is constitutively expressed regardless of the oxygen concentration, whereas the HIF-1α protein is rapidly broken down by ubiquitination and proteosomal degradation under normoxic conditions (9). Under hypoxic conditions, HIF-1α is stabilized and translocates from the cytoplasm to the nucleus where it dimerizes with HIF-1β, and the HIF-1 complex becomes transcriptionally activated. HIF-1 induces the transcription of a wide variety of genes, including those involved in glycolysis, angiogenesis, hematopoiesis, survival pathways, and invasion.

The overexpression of HIF-1α has been reported in many tumor types, including colon, breast, gastric, lung, skin, ovarian, pancreatic, prostate and renal carcinomas (10). A correlation between HIF-1α expression and tumor oxygenation was found in cervical carcinomas (11,12). Significant associations between HIF-1α overexpression and patient mortality have been shown in many different cancers, including those of the brain, breast and cervix (13–15).

HIF-1 is necessary for tumor growth. The disruption of HIF-1α by the intratumoral injection of small-interfering RNA (siRNA) was reported to result in the regression of human glioma xenografts (16), and in the tumor stasis of human cervical cancer and colon cancer xenografts (17). Furthermore, downregulation of HIF-1α by siRNA increased the sensitivity of cancer cells to chemotherapies (18,19) and irradiation (20). These studies indicate that HIF-1 also plays important roles in resistance to therapies.

In the present study, we show that the chemical compound ER-400583-00 is a novel HIF-1 inhibitor. We investigated the mode of action, pharmacodynamics, and antitumor activity of ER-400583-00. Our findings revealed that ER-400583-00 suppressed the proliferation of cancer cells most prominently in areas distal to the region of blood perfusion, where HIF-1α-expressing hypoxic cancer cells were located.

## Materials and methods

### Cells and compounds

Human U251 glioma cells were purchased from the Riken Cell Bank (Ibaraki, Japan). U251/vascular endothelial growth factor (VEGF)-placental alkaline phosphatase (PLAP) cells harbor a plasmid in which the PLAP reporter gene is under the control of a VEGF promoter, as described previously (21). The cells were cultured in Dulbecco's modified Eagle's medium supplemented with 10% fetal bovine serum, 100 U/ml penicillin, and 100 μg/ml streptomycin. The cells were incubated at 37°C in a humidified atmosphere containing 5% CO_2_ and either 21% O_2_ (normoxic conditions) or 2% O_2_ (hypoxic conditions).

ER-400583-00 was synthesized at Eisai Co., Ltd (Tokyo, Japan). For the cellular assays, ER-400583-00 was prepared in dimethyl sulfoxide (DMSO) and then diluted in the culture medium. The final concentration of DMSO in any incubation mixture did not exceed 0.2% (v/v). For the animal studies, ER-400583-00 was formulated in DMSO/Tween-80 (35:65), which was diluted 5-fold with saline immediately before administration.

### Cell-based HIF-1 reporter assay

HIF-1 reporter activity was determined as previously described (21). Briefly, U251/VEGF-PLAP cells were seeded onto 96-well plates at 4.0×10^4^ cells/well. After overnight incubation, the test compounds were added. The plates were incubated under hypoxic conditions for an additional 18 h. PLAP activity in the culture supernatant was then determined.

### Quantitative polymerase chain reaction (qPCR)

Total RNA was obtained using the RNeasy Mini kit (Qiagen Inc., Valencia, CA) and reverse transcription was performed using the High Capacity cDNA Reverse Transcription kit (Life Technologies, Carlsbad, CA). qPCR was carried out using the ABI7900HT Detection System and the TaqMan Universal PCR Master Mix (Life Technologies). The following TaqMan Gene Expression assays (Life Technologies) were used: Hs00153153_m1 (HIF-1α), Hs00154208_m1 [carbonic anhydrase IX (CA9)], Hs00173626_m1 (VEGFA), Hs00197884_m1 [solute carrier family 2, member 1 (SLC2A1)], Hs99999904_m1 [peptidylprolyl isomerase A (PPIA)], Hs99999907_m1 [β-2-microglobulin (B2M)], and Hs99999901_s1 (18S rRNA).

### HIF-1α enzyme-linked immunosorbent assay (ELISA)

The HIF-1α protein concentration in the samples was determined by a sandwich ELISA using the Human/Mouse Total HIF-1 alpha DuoSet IC (R&D Systems, Minneapolis, MN) according to the manufacturer's recommended protocol. Briefly, cells in 96-well plates were solubilized in 100 μl lysis buffer [50 mM Tris (pH 7.5), 300 mM NaCl, 10% glycerol, 3 mM EDTA, 1 mM MgCl_2_, 20 mM β-glycerophosphate, 25 mM NaF, 1% Triton X-100, and 1 mM Na_3_VO_4_). The lysates were diluted 5-fold with PBS containing 1% bovine serum albumin, and a 100 μl sample was used for the sandwich ELISA.

### Human tumor xenograft model

U251 cells were injected subcutaneously into the flank or hind leg of female BALB/cA nu/nu athymic mice, and allowed to grow to ~300 mm^3^. Tumor volumes were determined as length × (diameter)^2^/2, where length was the longest dimension and diameter was the shortest dimension. Local irradiation (10 Gy) was delivered to the tumor xenografts using the MBR-1520R-3 system (Hitachi, Tokyo, Japan) and a lead cage that shielded the whole body except for the tumor-bearing hind limb. All experiments were approved by the Animal Care and Use Committee at the Eisai Tsukuba Research Laboratories (Ibaraki, Japan).

### Pharmacodynamic studies

Mice bearing U251 tumor xenografts received a 10 ml/kg single oral administration of ER-400583-00. At various time points, animals were sacrificed and the tumors were removed, frozen in liquid nitrogen, and fragmented using a Multi-beads Shocker (Yasui Kikai, Osaka, Japan). To determine the amount of HIF-1α present, the fragmented tumors were lysed in lysis buffer [50 mM Tris (pH 7.5), 300 mM NaCl, 10% glycerol, 3 mM EDTA, 1 mM MgCl_2_, 20 mM β-glycerophosphate, 25 mM NaF, 1% Triton X-100, and 1 mM Na_3_VO_4_) and centrifuged at 1500 × g for 5 min. The HIF-1α concentration in the supernatant was determined by HIF-1α ELISA, as described above. The total protein concentration of the sample was determined by the BCA Protein assay kit (Thermo Fisher Scientific, Waltham, MA), and the HIF-1α protein concentration in each sample was normalized to the total protein concentration. For gene-expression analysis, the fragmented tumors were lysed in TRIzol LS Reagent (Life Technologies), and total-RNA was purified according to the manufacturer's instructions. qPCR was conducted as described above.

### Antitumor studies

Mice bearing U251 xenografts were stratified into groups comprising five animals with approximately equal mean tumor volumes. The animals received a 10 ml/kg oral administration of ER-400583-00 or vehicle once daily for 11 days. The tumor volumes were measured twice weekly, and the relative tumor volumes (the ratio of tumor volume to initial size before treatment) were calculated.

For combination studies of radiation therapy and ER400583-00, U251 tumor xenografts were allowed to grow on both hind legs of the mice. The mice were stratified into two groups of five animals. The tumor xenografts on the left hind leg of each mouse were locally irradiated on Day 0. The animals received an oral administration of ER-400583-00 or vehicle once daily for the following 4 days.

### Immunohistochemistry and image acquisition

For immunohistochemical analysis, mice bearing U251 tumor xenografts received an intraperitoneal injection of 5-bromo-2-deoxyuridine (BrdU, Sigma-Aldrich) as a 5 mg/ml solution in PBS containing 0.007 N HCl at 50 mg/kg. After 6 h, the mice received an intraperitoneal injection of pimonidazole (Hypoxyprobe-1 Plus kit; Millipore Corporation, Billerica, MA) as a 10 mg/ml solution in saline at 60 mg/kg. After 40 min, the mice were injected intravenously with 1 mg Hoechst 33342 fluorochrome as a 10 mg/ml solution in water (Invitrogen). After 20 min, the mice were sacrificed and the tumors were excised. They were embedded in Tissue-Tek O.C.T. Compound (Sakura Finetechnical, Nagano, Japan) in cryomolds, and stored at −80°C until sectioning.

The tumor cryosections were cut and air-dried for 5 min. Hoechst 33342 fluorescence images of entire tumor sections were captured using an Axiovert 200M fluorescence microscope (Carl Zeiss, Oberkochen, Germany) with a digital camera (Axiocam MRm, Carl Zeiss) at a resolution of 9.7 μm/pixel for HIF-1α and BrdU staining, and 2.4 μm/pixel for pimonidazole staining. The AxioVision digital image processing software (Carl Zeiss) was used for capturing the images. After image acquisition, the slides were rinsed in water, fixed in 1% paraformaldehyde (PFA) in PBS for 10 min, rinsed twice in PBS, and then stained for HIF-1α, BrdU, or pimonidazole.

HIF-1α was detected using a rabbit polyclonal antibody NB100-449B (Novus Biologicals, Littleton, CO) at a 1:500 dilution with a horseradish peroxidase-conjugated avidin-biotin detection system (Vectastain Elite ABC kit, Vector Laboratories, Burlingame, CA) and diaminobenzidine (DAB, Muto Pure Chemicals, Tokyo, Japan). Slides were counterstained with hematoxylin, and mounted using Aquatek (Merck & Co., Inc.; Whitehouse Station, NJ). Whole-slide red, green and blue (RGB) images were obtained using an AP-200 slide imager (Kurabo Industries Ltd., Osaka, Japan) at a resolution of 1.7 μm/pixel. Pimonidazole was detected using a fluorescein isothiocyanate (FITC)-labeled hypoxyprobe-1 Mab1 monoclonal antibody (Hypoxyprobe-1 Plus kit) according to the manufacturer's instructions.

Slides were mounted using Vectashield (Vector Laboratories). FITC fluorescence images of entire tumor sections were obtained using an Axiovert 200M fluorescence microscope at a resolution of 2.5 μm/pixel. For BrdU detection, slides were treated with 50% formamide in 2X SSC at 60°C for 3 h. The slides were then rinsed twice in 2X SSC, treated with 2 N HCl at 37°C for 30 min, neutralized twice in 0.1 M sodium borate, and rinsed twice in PBS. BrdU was detected using the rat monoclonal antibody OBT0030 (Accurate Chemical & Scientific Corporation, Westbury, NY) at a 1:500 dilution with the horseradish peroxidase-conjugated avidin-biotin detection system (Vectastain Elite ABC kit, Vector Laboratories) and DAB (Muto Pure Chemicals). Slides were counterstained with hematoxylin and mounted using Aquatek (Merck, Inc.). Whole-slide RGB images were obtained using the AP-200 slide imager (Kurabo Industries, Ltd.) at a resolution of 1.7 μm/pixel.

### Image analysis

The fractions of tissue that were positive for HIF-1, pimonidazole, and BrdU at each measured distance from the region of blood perfusion were determined as follows. Initially, Photoshop Elements software (Adobe Systems Inc., San Jose, CA) was used to enlarge the Hoechst 33342 fluorescence images and to overlay them with the corresponding images of HIF-1α, pimonidazole, or BrdU staining. After the removal of necrotic tissue, skin, and staining artifacts, three binary images (denoted Perfusion, Signal and Tissue) were produced for each layered image.

The Perfusion images were produced by binarizing the Hoechst 33342 fluorescence images with the threshold value fixed in each experiment. The signal images for HIF-1α and BrdU were produced from the RGB images by selecting all pixels within the color range for DAB, which was determined separately for each experiment. The Signal images for pimonidazole were produced by binarizing the FITC fluorescence images with a threshold that was determined separately for each image, which produced a pimonidazole-positive fraction of 1% in the Hoechst 33342-positive regions. The Tissue images for HIF-1α and BrdU were produced from the RGB images by selecting all pixels with RGB values below or equal to 200. The Tissue images for pimonidazole were produced by manual delineation of the tumor boundaries. Photoshop Elements software was used for binarizing the fluorescence images, and Lumina Vision software (Mitani Corporation, Fukui, Japan) was used for binarizing the RGB images.

All binary images for the HIF-1 and BrdU analysis were reduced to 25% of the original size, and those for the pimonidazole analysis were reduced to 50% of the original size. The distance from each positive pixel in the Signal and Tissue images to the nearest positive pixel in the corresponding Perfusion image was then measured using JAVA software programmed by the authors. The data were tabulated and the fraction of Signal-positive pixels at each distance from the area of perfusion was determined by dividing the number of pixels in the Signal image at each distance by the number of pixels in the Tissue image at the same distance.

## Results

### Discovery of ER-400583-00 as a small-molecule HIF-1α inhibitor

**Figure 1:**
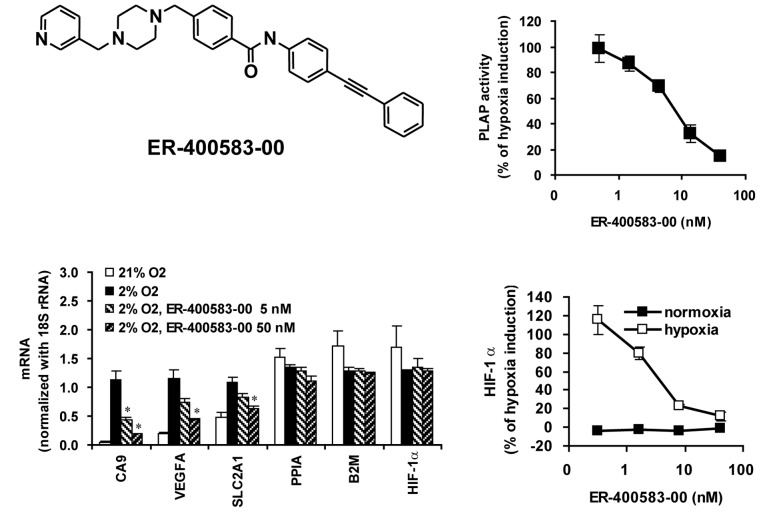
ER-400583-00 inhibits hypoxic induction of HIF-1 activity. (A) Chemical structure of ER-400583-00. (B) ER-400583-00 inhibited the hypoxic induction of U251/VEGF-PLAP reporter activity. Results are expressed as the percentage of PLAP activities induced under hypoxic conditions. Data points represent the mean of five independent experiments; bars indicate the SD. (C) Effect of ER-400583-00 on gene expression. U251 cells (6×10^5^ cells/well in 6-well plates) were incubated under the indicated conditions for 6 h, then mRNA levels were measured by qPCR and normalized to 18S rRNA. Data points represent the mean of three wells; bars indicate the SD, ^*^P<0.01 compared with 2% O_2_. (D) ER-400583-00 suppressed the hypoxic induction of HIF-1α protein accumulation. U251 cells (4×10^4^ cells/well in 96-well plates) were incubated with different concentrations of ER-400583-00 under normoxic or hypoxic conditions for 6 h, then the amounts of HIF-1α protein were measured by an ELISA. Data points represent the mean of three wells; bars represent the SD.

In order to search for novel small molecules that suppressed the HIF-1 pathway, we generated a human glioma cell line expressing a PLAP reporter gene under the control of a VEGF promoter, which contained the active HIF-1 binding site (U251/VEGF-PLAP). The reporter activity was increased more than 10-fold when the cells were cultured under hypoxic conditions. U251-based HIF-1 reporter assay has also been demonstrated by another group (22). We performed high-throughput screening of a small-molecule library of 43,000 compounds using U251/VEGF-PLAP. Through derivatization of a hit compound, we discovered ER-400583-00 (Fig. 1A), which inhibited the induction of HIF-1 reporter activity in response to hypoxia, with a half-maximal inhibitory concentration (IC_50_) value of 7.9 nM (Fig. 1B).

To examine whether ER-400583-00 suppressed the induction of endogenous HIF-1 target genes in response to hypoxia, U251 cells were cultured under normoxic or hypoxic conditions, with or without ER-400583-00, and gene expression was examined. The HIF-1 target genes CA9, VEGFA, and SLC2A1 were induced by hypoxia (Fig. 1C). ER-400583-00 suppressed the hypoxia-induced accumulation of the messenger RNAs (mRNAs) of these HIF-1 target genes. By contrast, ER-400583-00 did not affect the mRNA levels of the housekeeping genes PPIA and B2M, or those of HIF-1α. The amount of HIF-1α protein present was measured. The mean ± SD HIF-1α levels in U251 cells incubated at 4×10^4^ cells/well in 96-well plates under normoxic and hypoxic conditions for 6 h were 37.8±13.4 pg/ml and 367.2±33.0 pg/ml, respectively. Incubation under hypoxic conditions for 6 h thus resulted in a 9.7-fold increase of the amount of HIF-1α protein in U251 cells. ER-400583-00 did not reduce the amount of HIF-1α protein present in U251 cells under normoxic conditions. However, it suppressed the induction of HIF-1α protein in response to hypoxia, with an IC_50_ value of 3.7 nM (Fig. 1D).

### Pharmacodynamics and antitumor activity of ER-400583-00 in a human tumor xenograft model

**Figure 2:**
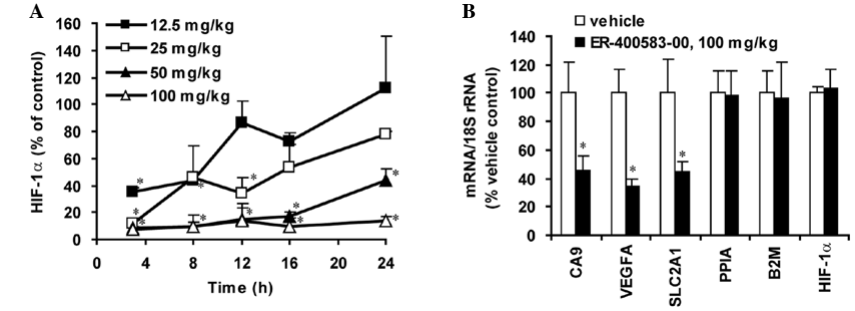
Pharmacodynamic profiles of ER-400583-00. Mice bearing U251 tumor xenografts were treated with a single dose of ER-400583-00 at the indicated dosage. (A) Tumors were harvested at various time points as indicated. The amounts of HIF-1α in the tumors were measured by an ELISA and expressed as a percentage of the vehicle control. Data points represent the mean of three animals, ^*^P<0.01 compared with the vehicle control; bars represent the SD. (B) Tumors were harvested 24 h after dosing. mRNA levels of the indicated genes were measured by qPCR, normalized to the 18S rRNA level, and expressed as a percentage of the vehicle control. Columns represent the mean of six animals, ^*^P<0.01 compared with the vehicle control; bars represent the SD.

Next, we investigated the pharmacodynamics of ER-400583-00 using a human U251 tumor xenograft model in athymic mice. ER-400583-00 was administered orally as a single dose, and the amount of HIF-1α present in tumors was determined over time. A significant decrease in the amount of HIF-1α was observed in tumors as early as 3 h after the administration of ER-400583-00 (Fig. 2A). The amount of HIF-1α returned to the control level 12 h after the administration of ER-400583-00 at a dose of 12.5 mg/kg, but the effect was sustained for longer at a higher dosage. ER-400583-00 at a dose of 100 mg/kg decreased the amount of HIF-1α to less than 10% of that in control tumors 3 h after administration, and the suppressive effect was sustained for 24 h. qPCR analysis revealed that the administration of ER-400583-00 at 100 mg/kg significantly decreased mRNA levels of the HIF-1 target genes CA9, VEGFA, and SLC2A1 24 h after treatment (Fig. 2B). By contrast, the mRNA expression levels of HIF-1α, and the house-keeping genes PPIA and B2M, were not altered.

**Figure 3:**
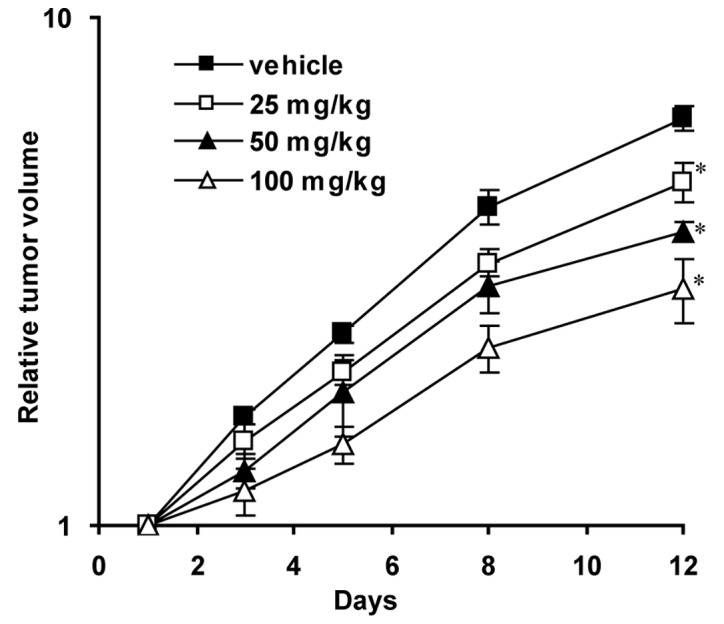
ER-400583-00 suppressed U251 tumor xenograft growth. Mice bearing U251 tumor xenografts received oral administration of ER-400583-00 or vehicle daily for 11 days. The y-axis indicates relative tumor volume in log scale. Data points represent the mean of five animals, ^*^P<0.05 compared with the vehicle control; bars represent the SD.

We then evaluated the antitumor activity of ER-400583-00 in human U251 tumor xenografts. The daily oral administration of ER-400583-00 significantly delayed tumor growth, as assessed by changes in tumor volume (Fig. 3).

### HIF-1α expression and hypoxia in tumors

**Figure 4:**
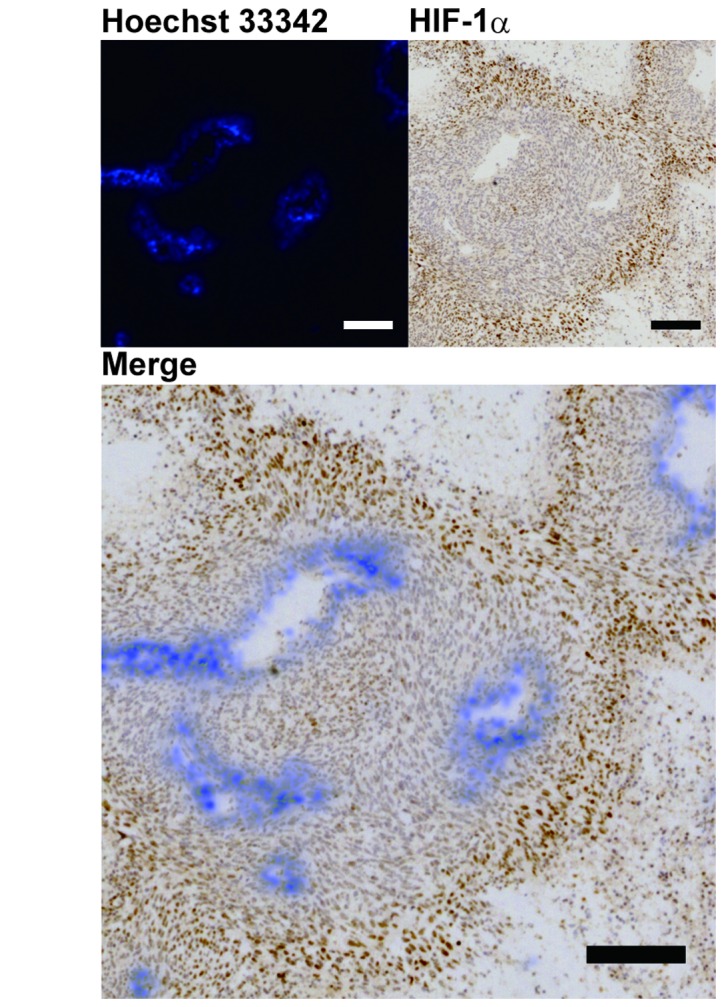

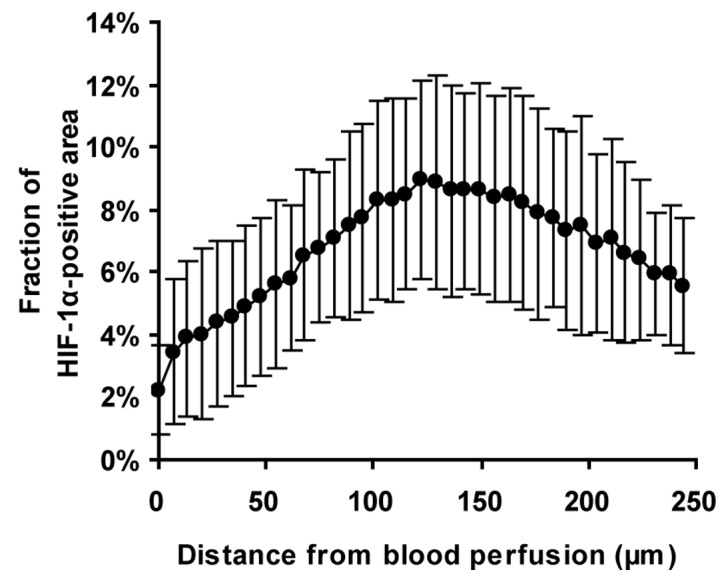

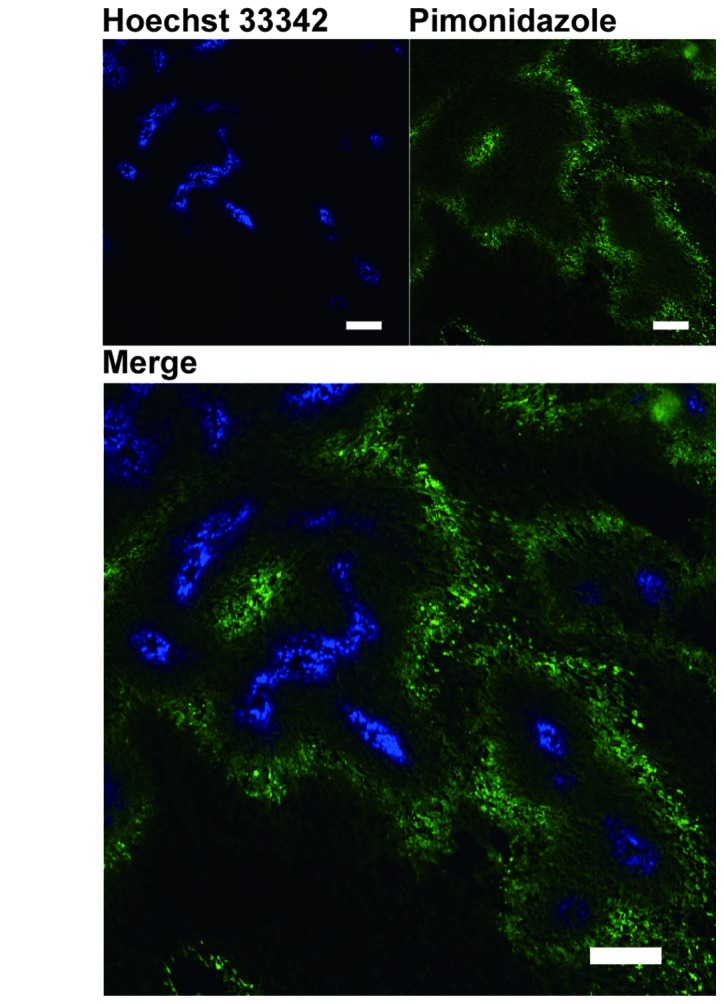

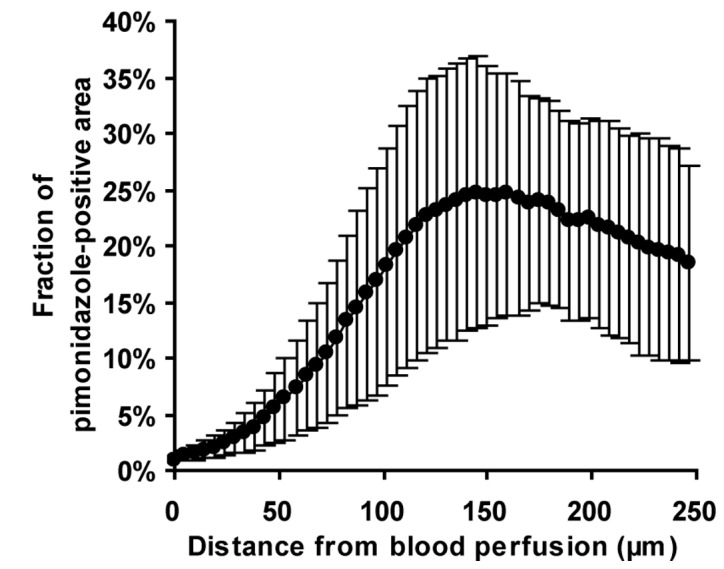
Distributions of HIF-1α and hypoxia in U251 xenograft tumors relative to the region of blood perfusion. (A and C) Hoechst 33342 fluorescent images (blue) were digitally overlaid onto images of immunohistochemical staining for either HIF-1β (brown, A) or pimonidazole (green, C). Bar, 200 μm. (B) Line graphs show the fractions of HIF-1α and (D) pimonidazole labeling at each distance from the nearest region of blood perfusion. Entire tumor sections were analyzed. Data points represent the mean of three tumors; bars represent the SD.

We examined the distributions of HIF-1α expression and hypoxia in conjunction with blood perfusion in U251 tumor xenografts, in order to determine whether ER-400583-00 exerted antitumor activity against HIF-1α-expressing hypoxic cancer cells. Blood perfusion was assessed using the distribution of Hoechst 33342 fluorochrome, which has limited tissue penetration (23). Tumor sections were then stained for HIF-1α immunohistochemistry and the images for both signals were merged. The results showed that cancer cells with higher HIF-1α expression were located distal to the region of blood perfusion (Fig. 4A).

Quantitative analysis of the merged images confirmed that the size of the HIF-1α-positive area increased with distance from the region of blood perfusion, and reached a maximum at a distance between 100 and 200 μm (Fig. 4B). The distribution of hypoxia was also analyzed by a similar method, using pimonidazole (23,24) as a marker, and appeared comparable to that of HIF-1α (Fig. 4C). Quantitative analysis revealed that the pimonidazole-positive area also increased with distance from the region of blood perfusion, and reached a maximum at a distance between 100 and 200 μm (Fig. 4D). These results indicated that, in U251 tumor xenografts, hypoxic tissues were located distal to the region of blood perfusion and that HIF-1α was induced in cancer cells in this region.

### Antitumor activity of ER-400583-00 against a tumor hypoxic region

**Figure 5:**
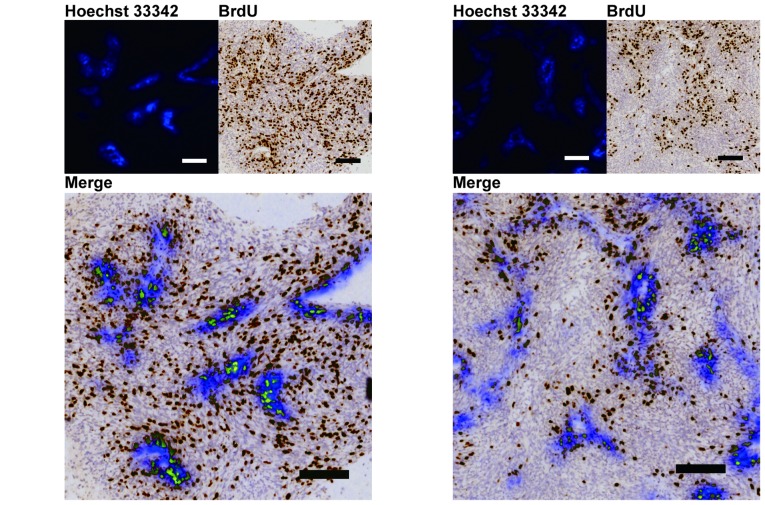

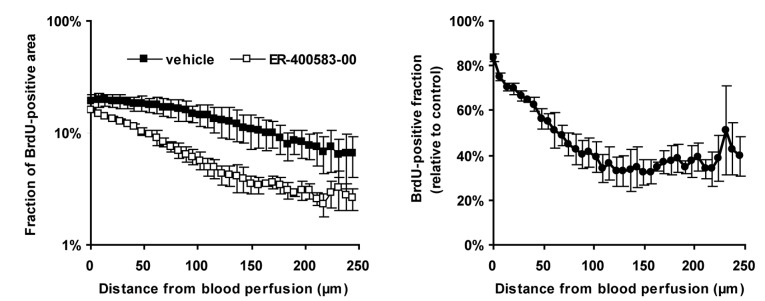
ER-400583-00 suppressed cell proliferation distal to the region of blood perfusion in U251 xenograft tumors. Mice bearing U251 tumors were orally treated with either vehicle or 100 mg/kg ER-400583-00 once daily for 3 days. Tumors were collected 24 h after the last treatment. Hoechst 33342 fluorescent images of tumor cryosections were digitally captured, and overlaid onto images from subsequent immunohistochemical staining for BrdU. (A) Representative composite image of a vehicle control tumor and (B) an ER-400583-00-treated tumor. Hoechst 33342 is shown in blue and BrdU in brown. Bar, 200 μm. (C) The fraction of BrdU labeling at each distance from the nearest region of blood perfusion. Entire tumor sections were analyzed. Data points represent the mean of four tumors; bars represent the SD. (D) The ratio of the BrdU-positive fraction in the ER-400583-00-treated tumors relative to the vehicle control tumors. Data points represent the mean of four tumors; bars represent the SD.

The distributions of proliferating cells were analyzed using BrdU staining, in order to examine whether ER-400583-00 acted against HIF-1α-expressing cancer cells in the hypoxic region of U251 tumor xenografts. In ER-400583-00-treated tumors, cancer cells in areas distal to the region of blood perfusion appeared to be less proliferative than those in control tumors (Fig. 5A and 5B).

Quantitative analysis revealed that, in control tumors, the values of proliferation indices decreased as the distance from the region of blood perfusion increased (Fig. 5C). In ER-400583-00-treated tumors, cancer cells near the region of blood perfusion showed similar proliferative activity to those in control tumors. However, the values of proliferation indices decreased more notably as the distance increased (Fig. 5C), and fell below 50% of those in control tumors in tissues at a distance of 100 μm from the region of blood perfusion (Fig. 5D). These results indicated that the growth-suppressive effect of ER-400583-00 was more prominent in HIF-1α-expressing hypoxic cancer cells than in non-hypoxic cancer cells.

**Figure 6:**
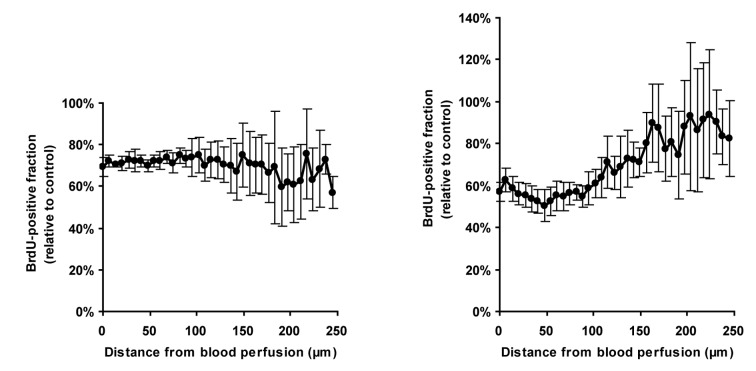
Hypoxic cancer cells distal to the region of blood perfusion in U251 xenograft tumors are resistant to radiation therapy. (A) U251 tumor xenografts were locally irradiated and collected at 2 days and (B) 7 days post-irradiation. The ratio of the BrdU-positive fraction in irradiated tumors relative to control tumors is shown. Data points represent the mean of four tumors; bars represent the SD.

Hypoxic cancer cells have been reported to be radiation-resistant (25). To determine whether hypoxic cancer cells in U251 tumor xenografts were also resistant to radiation therapy, we analyzed the pattern of BrdU incorporation in tumor cells after irradiation. The values of proliferation indices were slightly decreased, regardless of the distance from the region of blood perfusion, at 2 days post-irradiation (Fig. 6A). At 7 days post-irradiation, the values of proliferation indices in tissues near the region of blood perfusion remained low (Fig. 6B), whereas those in tissues distal to the region of blood perfusion had recovered to control levels. These results indicated that the hypoxic cancer cells in U251 tumor xenografts were resistant to radiation therapy.

### Combined antitumor activity of ER-400583-00 and radiation therapy

**Figure 7:**
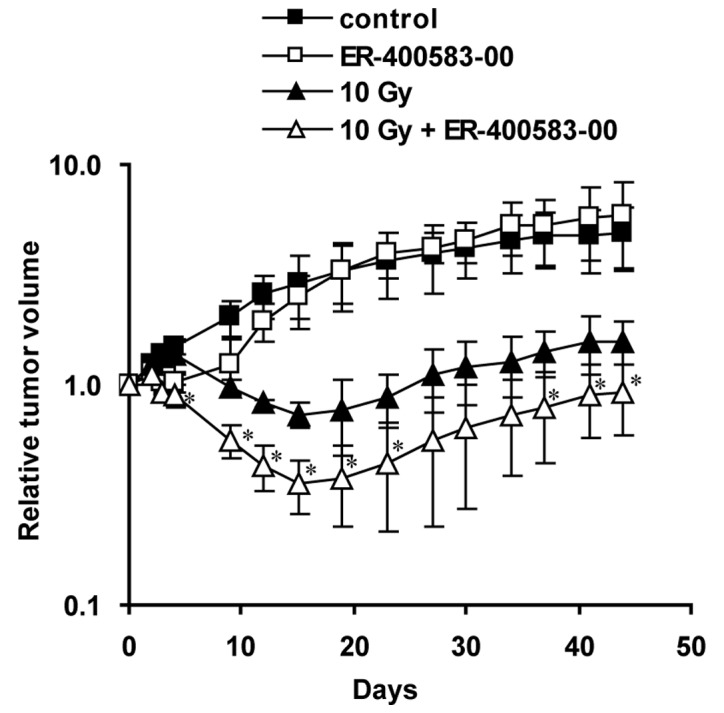
Combined antitumor activity of ER-400583-00 and radiation therapy. Mice bearing U251 tumor xenografts were locally irradiated on Day 0, and received oral administration of ER-400583-00 or vehicle once daily for the subsequent 4 days. Data points represent the mean of five animals, ^*^P<0.05 compared with the radiation-treated group; bars represent the SD.

Finally, we evaluated the combined antitumor activity of ER-400583-00 and radiation therapy in the U251 tumor xenograft model (Fig. 7). In the radiation-treated group, the tumors had shrunk by 4 days after irradiation, and reached a minimum size at 15 days post-irradiation. In the combined-treatment group, the tumors shrank more rapidly and the minimum tumor size was significantly lower than that of the radiation-treated group. A synergistic interaction was observed at 15 days post-irradiation.

## Discussion

This study identified ER-400583-00 as a novel small-molecule HIF-1 inhibitor. ER-400583-00 suppressed the accumulation of HIF-1α protein under hypoxic conditions but did not affect HIF-1α mRNA levels, indicating that it acted on either the translation or degradation of the HIF-1α protein. HIF-1α is degraded by a proteasome-dependent pathway in normoxic cells. Under normoxic conditions, HIF-1α is proline hydroxylated by specific proline hydroxylases (26). This hydroxylation promotes the binding of HIF-1α to the von Hippel Lindau E3 ligase complex, which is followed by ubiquitination and proteasomal degradation. Iron chelators such as deferoxamine and cobalt inhibit the proline hydroxylases and induce HIF-1α accumulation under normoxic conditions (27). Although ER-400583-00 suppressed the HIF-1α protein accumulation induced by hypoxia, it did not suppress the HIF-1α accumulation induced by deferoxamine (data not shown); this indicated that ER-400583-00 enhanced the degradation of HIF-1α under hypoxic conditions through a proline hydroxylation-dependent ubiquitination pathway. Further studies are needed to clarify the precise mechanism of ER-400583-00 action.

Our histological analysis revealed that cells located at a distance between 100 and 200 μm away from blood perfusion were pimonidazole-positive in U251 xenograft tumors. Tissues far away (>200 μm) were necrotic and pimonidazole-negative. This distance is well consistent with the reported limitation of oxygen diffusion (28). The main pathogenetic mechanisms involved in the development of hypoxia are related to diffusion, anemia, or perfusion (2). Anemia was not observed in this model (data not shown). Our histological analysis showed that most platelet/endothelial cell-adhesion molecule 1 (CD31)-positive vessels stained positive for Hoechst 33342 fluorochrome, and that pimonidazole staining was not observed near the vessels. These data indicated that most vessels in this model were functionally normal and well-perfused. In contrast to perfusion-related hypoxia, which occurs abruptly and intermittently, diffusion-related hypoxia is chronic. The chronic nature of the hypoxia in this model facilitated the analysis of differences in cellular responses among local microenvironments.

Although hypoxia is a strong inducer of HIF-1α, other factors such as growth signals and lactate accumulation have been reported to induce this protein even under normoxic conditions. We compared the amounts of HIF-1α in several cancer cell lines, and found that U251 cells had relatively low expression levels under normoxic conditions (data not shown); this indicated that factors other than hypoxia were less effective at inducing HIF-1α in U251 cells. The distribution of HIF-1α in U251 xenograft tumors was well correlated with the distribution of hypoxia. These data indicated that hypoxia was a dominant inducer of HIF-1α in U251 xenograft tumors.

We found that ER-400583-00 suppressed the proliferation of cancer cells distal to the region of blood perfusion without affecting those near the vasculature in U251 xenograft tumors. This indicated that the cellular response to HIF-1 blockade differed under different microenvironments. Hypoxic conditions did not enhance the growth-inhibitory activity of ER-400583-00 *in vitro* (data not shown). These data are consistent with the results of previous studies showing that a loss of HIF-1α had little effect on *in vitro* cell growth under hypoxic conditions (29,30). Dominant-negative HIF-1α was shown to suppress pancreatic cancer cell growth under hypoxic and glucose-deprived conditions (31). Nutrients such as glucose and amino acids are also transported by the blood, and are likely to be scarce in areas distal to a region of blood perfusion. These factors might enhance the growth-inhibitory activity of ER-400583-00 in such areas.

ER-400583-00 augmented the antitumor effect of radiation therapy in the U251 xenograft model. Our immunohistochemical analysis revealed that hypoxic cancer cells were resistant to radiation therapy in this model, and that ER-400583-00 showed antitumor activity against them. Selective cell killing by poly (ADP-ribose) polymerase 1 (PARP1) inhibitors was reported recently (32). In this study, the authors showed that PARP inhibitor-treated xenografts displayed decreased clonogenic survival following experimental radiotherapy. We hypothesized that the enhancement of antitumor activity by ER-400583-00 was also achieved by targeting hypoxic cancer cells. In our preliminary experiments, hypoxic cancer cells in the U251 xenograft model were also resistant to cytotoxic agents such as camptothecin-11 (CPT-11) and paclitaxel. Combining ER-400583-00 with these drugs could therefore be a promising approach, and we plan to evaluate this combined antitumor activity in future studies.

In summary, this study discovered the orally active, novel HIF-1 inhibitor ER-400583-00. Our results showed that treatment with ER-400583-00 achieved sustained HIF-1α suppression in xenograft tumors in animal studies. ER-400583-00 exhibited enhanced cytotoxicity against hypoxic cancer cells in tumors and enhanced antitumor activity in combination with radiation therapy.
